# HIV/AIDS-related knowledge awareness and risk behaviors among injection drug users in Maanshan, China: a cross-sectional study

**DOI:** 10.1186/s12889-016-2786-6

**Published:** 2016-02-01

**Authors:** Baifeng Chen, Yu Zhu, Rui Guo, Shushu Ding, Zhen Zhang, Huaying Cai, Hongbin Zhu, Yufeng Wen

**Affiliations:** 1 School of Public Health, Wannan Medical College, 22 West Wenchang Road, Wuhu, Anhui Province 241002 China; 2 Centre for Disease Control of Maanshan City, 849 Jiangdong Dadao, Maanshan, Anhui Province 241000 China

**Keywords:** HIV/AIDS, Injection drug user, Syringe sharing, Condom use

## Abstract

**Background:**

Unsafe injection practices significantly increase the risk of human immunodeficiency virus (HIV) infection among injection drug users (IDUs). Little is known about how demographic characteristics of IDUs are linked to HIV-related risk behaviors in the central regions of China.

**Methods:**

A cross-sectional survey was conducted at Mandatory Detoxification Centers (MDCs) and the community in Maanshan, China.

**Results:**

Of the 916 IDUs, 96.4 % reported a history of heroin use during the past year, 93.4 % had HIV/AIDS knowledge, 16.8 % reported receptive syringe sharing and 12.2 % reported inconsistent condom use in commercial sex in the past year. Unsafe injection practice was associated with increased odds of minority ethnicity, lower level of education, and no peer education in the past year. Unsafe sex practice was associated with increased odds of being single, 18–30 years of age, non-local residence, and history of methamphetamine use in the past year.

**Conclusions:**

Integrated interventions to promote safe injection and protected commercial sex practices targeting IDUs must also consider individual and socio-environmental factors.

## Background

Since the earliest evidence of the prevalence of AIDS among injection drug users (DIUs) was seen in China in 1989 [1], the injection of drugs has become one of the primary channels of HIV infection in China. Findings from geospatial analysis of HIV subtypes, behavioral surveys among IDUs and their sex partners and routine surveillance activities indicated that syringe sharing among IDUs, and sexual contact between IDUs and sex workers, were the major drivers of later sub-epidemics across mainland China [2].

Research has shown that 72.5 % of individuals dependent on heroin in China were injecting, and their syringe sharing rate was up to 69.5 % [3]. By the end of 2011, 780,000 people were estimated to be living with HIV/AIDS in China, and the number of people infected with HIV via injecting drug use was estimated at about 221,000 [4]. Of the estimated 14.0 million people who inject drugs worldwide, the United Nations Office on Drugs and Crime (UNODC) estimates that 1.6 million are living with HIV. The Russian Federation, the United States, and China account for one half of the global number of IDUs living with HIV (21 %, 15 %, and 10 %, respectively) [5].

IDUs are at high risk for HIV and other blood-borne infections due to the sharing of contaminated needles and syringes. It is reported that the HIV epidemics in several other Asian countries started in IDUs and spread via syringe sharing and unprotected sex with sex workers to the general population [6]. There is often an overlap between communities of IDUs and communities of sex workers in many regions of Asia, as those who sell sex may do it to fund a drug habit, or they may have been involved in sex work first before turning to drug use [7].

Drug users in China are treated in MDCs or in community-based treatment programs. MDCs are administered by the Ministry of Public Security and function as penitentiary, reeducation-through-labor camp, and detoxification centers for repeat offenders of drug-related charges. In the past, MDCs have come under harsh scrutiny for human rights abuses, including failure to treat drug-addiction withdrawal symptoms as well as other general medical problems. Some researchers and human rights advocates have argued that living conditions in MDCs actually lead to relapse and risky behaviors [8].

Currently, the Chinese government is taking various measures to prevent and control the transmission of AIDS among IDUS. IDUs in China were mainly concentrated in large cities and border regions (eg. Beijing, Shanghai, Yunnan). With the spread of drug use across the country, small cities like Maanshan have more IDUs in recent years, although little is known about the relationship between demographic characteristics and HIV/AIDS-related knowledge awareness and risk behaviors among IDUs in Maanshan. Therefore, we conducted a cross-sectional study to identify the personal profiles and risk behaviors among IDUs from the MDC and community in Maanshan city to better understand the characteristics of IDUs and to promote safe behaviors in this high-risk population. These preliminary findings provide evidence for better implementation of targeted intervention measures and strategies for IDUs.

## Methods

### Study Population and Recruitment

A cross-sectional study was conducted between April 2013 and September 2014 to analyze the factors associated with HIV/AIDS knowledge awareness and risk behaviors among IDUs recruited from the MDCs and community in Maanshan. There were 168 IDUs in the Maanshan MDCs during our study period, of which 155 people were selected to participate in the current study. With the help of methadone clinics of Maanshan’s Center for Disease Control and Prevention (CDC), 63 IDUs from 63 different communities were assigned as seeds, they assisted us in recruiting IDUs to participate and refer fellows through snowball sampling, a total of 761 IDUs were selected to participate in our investigation.

Individuals were invited to participate if they were 18–60 years old, had a history of at least one drug injection in the previous year, not previously diagnosed with HIV, willing to provide informed consent to participate in the survey and provide a blood sample for HIV testing. Eligible participants were notified of potential risks and benefits of study participation and understood that participation was voluntary. Written informed consent was obtained from all subjects who were eligible and willing to participate. Subjects were given 30 RMB (about 5 USD) for their participation, and the study was reviewed and approved by the Ethics committee of Wannan Medical College, China.

### Data Collection

Face-to-face interviews were conducted by well-trained staff members of the CDC in Maanshan, Interviews were conducted in on-site private rooms and the study protocol protected confidentiality through the use of personal identification numbers. The questionnaire was adapted from the National HIV Sentinel Surveillance embodiment and included participants’ demographic characteristics, HIV-related knowledge, sexual and drug-use behaviors, and peer education programs organized by local CDC. Eight HIV-related questions were used to assess HIV knowledge level, including three questions about HIV transmission modes, three questions about HIV misconceptions and two questions about HIV revention. All questions were weighted equally. Each correct answer was combined into an overall score from 0–8. Two groups were stratified according to whether they got a six or higher HIV-related knowledge score. HIV/AIDS knowledge awareness was defined as a total score greater than or equal to six points, otherwise defined as unawareness [9]. Participants were asked about the frequency and types of drugs used during the past year, injection was defined as intravenous, intramuscular, or subcutaneous. Receptive sharing of syringes was defined as having injected with a needle and syringe that someone else had previously used to inject. Participants were asked about the frequency of condom use if they reported commercial sex intercourse during the past year, and if they reported not always using condoms with any of their commercial sexual partners, they were classified as inconsistent condom users. Blood samples were collected from all eligible participants and used to assess the presence of HIV. Initial screening was conducted using ELISA method (ELISA-1) and confirmation tests were done on positive cases using a different ELISA method (ELISA-2). A result was considered positive only if the confirmation test was also positive.

### Statistical Analysis

Descriptive analysis was used to examine the demographic and behavioral characteristics of study participants. Univariate analysis and multivariate logistic regression models were used to identify factors related to HIV/AIDS knowledge awareness, receptive sharing of syringes, and inconsistent condom use. Variables that had a significance level of *p* < 0.10 in the univariate analysis were considered potential factors for inclusion in the multivariate models. Final multivariate models were determined by performing stepwise backward elimination to remove covariates not significant at the *P* < 0.05 level. The adjusted odds ratios (OR) and 95 % confidence intervals (CIs) are reported. Data entry was done in Epi Info 3.5. The database was then exported to SPSS 20.0 (SPSS Inc., Chicago, USA) for analysis. *P* < 0.05 was considered to be statistically significant.

## Results

### Characteristics of Participants

A total of 916 participants were recruited and completed the survey and HIV testing. Overall, three subjects were HIV-positive. The majority of participants were male (78.4 %) and of Han ethnicity (98.5 %). Most participants reported a history of heroin use (96.4 %) during the past year. A total of 856 (93.4 %) participants had HIV/AIDS knowledge, 154 (16.8 %) participants reported receptive sharing of syringes, and 112 (12.2 %) reported unprotected commercial sex in the past year. Characteristics of the participants are listed in Table 1.

**Table 1:** Socio-demographic and behaviors characteristics of the Study Participants (*n* = 916)

Socio-demographics and behavioral characteristics	n (%)
Recruitment methods	
Mandatory Detoxification Centers	155 (16.9)
Community	761 (83.1)
Gender	
Male	718 (78.4)
Female	198 (21.6)
Age (years)	
18–29	92 (10.0)
30–39	517 (56.4)
40–60	307 (33.5)
Marital status	
Single	186 (20.3)
Married or cohabiting	626 (68.3)
Divorced or widowed	104 (11.4)
Residential status	
Local resident	878 (95.9)
Non-local resident	38 (4.1)
Ethnicity	
Han	902 (98.5)
Minority ethnic	14 (1.5)
Education level	
≤Primary school	86 (9.4)
Junior high	585 (63.9)
≥Senior high	245 (26.7)
Drugs used in the past year	
Heroin	883 (96.4)
Methamphetamine	104 (11.4)
Other	29 (3.2)
Peer education in the past year	
Yes	498 (54.4)
No	418 (45.6)
HIV/AIDS knowledge awareness	
Yes	856 (93.4)
No	60 (6.6)
Receptive sharing of syringe in the past year	
Yes	154 (16.8)
No	762 (83.2)
Unprotected commercial sexual intercourse in the past year	
Yes	112 (12.2)
No (consistent condom use/never engage in commercial sex)	804 (87.8)

### Univariate Analysis

Table 2 presents the univariate analyses of potential associated factors related to HIV/AIDS knowledge awareness, receptive sharing of syringes and inconsistent condom use, respectively.

**Table 2:** Univariate analysis of the factors associated with HIV/AIDS knowledge awareness, receptive sharing of syringe, and inconsistent condom use

Socio-demographics and behavioral characteristics	HIV/AIDS knowledge awareness			Receptive syringe sharing in the past year			Condom use in the past year		
	Unknown (*n* = 60)	Known (*n* = 856)	OR (95 % CI)	Never (*n* = 762)	Yes (*n* = 154)	OR (95 % CI)	Consistent condom use/never engagein commercial sex (*n* = 804)	Inconsistent condom use in commercial sex (*n* = 112)	OR (95 % CI)
Recruitment methods									
Mandatory Detoxification Centers	10 (6.5)	145 (93.5)	1	119 (76.8)	36 (23.2)	1	131 (84.5)	24 (15.5)	1
Community	50 (6.6)	711 (93.4)	0.98 (0.49-1.98)	643 (84.5)	118 (15.5)	0.61 (0.40-0.92)**	673 (88.4)	88 (11.6)	0.71 (0.44-1.16)
Gender									
Male	50 (7.0)	668 (93.1)	1	587 (81.8)	131 (18.2)	1	627 (87.3)	91 (12.7)	1
Female	10 (5.1)	188 (94.9)	1.41 (0.70-2.83)	175 (88.4)	23 (11.6)	0.59 (0.37-0.95)**	177 (89.4)	21 (10.6)	0.82 (0.49-1.35)
Age (years)									
18-29	15 (16.3)	77 (83.7)	1	75 (81.5)	17 (18.5)	1	73 (79.3)	19 (20.7)	1
30-39	20 (3.9)	497 (96.1)	4.84 (2.38-9.86)***	428 (82.8)	89 (17.2)	0.92 (0.52-1.63)	450 (87.0)	67 (13.0)	0.57 (0.33-1.01)*
40-60	25 (8.1)	282 (91.9)	2.20 (1.10-4.37)**	259 (84.4)	48 (15.6)	0.82 (0.44-1.51)	281(91.5)	26 (8.5)	0.36 (0.19-0.68)**
Marital status									
Single	15 (8.1)	171 (91.9)	1	154 (82.8)	32 (17.2)	1	141 (75.8)	45 (24.2)	1
Married or cohabiting	36 (5.8)	590 (94.2)	1.44 (0.77-2.69)	522 (83.4)	104 (16.6)	0.96 (0.62-1.48)	584 (93.3)	42 (6.7)	0.23 (0.14-0.36)***
Divorced or widowed	9 (8.7)	95 (91.3)	0.93 (0.39-2.20)	86 (82.7)	18 (17.3)	1.01 (0.53-1.90)	79 (76.0)	25 (24.0)	0.99 (0.57-1.74)
Residential status									
Local resident	48 (5.5)	830 (94.5)	1	737 (83.9)	141 (16.1)	1	780 (88.8)	98 (11.2)	1
non-local resident	12 (31.6)	26 (68.4)	0.13 (0.06-0.26)***	25 (65.8)	13 (34.2)	2.72 (1.36-5.44)**	24 (63.2)	14 (36.8)	4.64 (2.33-9.27)***
Ethnicity									
Han	54 (6.0)	848 (94.0)	1	755 (83.7)	147 (16.3)	1	794 (88.0)	108 (12.0)	1
Minority ethnic	6 (42.9)	8 (57.1)	0.09 (0.03-0.25)***	7 (50.0)	7 (50.0)	5.13 (1.77-14.86)**	10 (71.4)	4 (28.6)	2.94 (0.91-9.54)*
Education level									
≤Primary school	16 (18.6)	70 (81.4)	1	60 (69.8)	26 (30.2)	1	71 (82.6)	15 (17.4)	1
Junior high	36 (6.2)	549 (93.8)	3.49 (1.84-6.61)***	486 (83.1)	99 (16.9)	0.47 (0.28-0.78)**	511 (87.4)	74 (12.6)	0.69 (0.37-1.26)
≥Senior high	8 (3.3)	237 (96.7)	6.77 (2.78-16.48)***	216 (88.2)	29 (11.8)	0.31 (0.17-0.57)***	222 (90.6)	23 (9.4)	0.49 (0.24-0.99)**
Drugs used in the past year									
Heroin									
No	5 (15.2)	28 (84.8)	1	29 (87.9)	4 (12.1)	1	30 (90.9)	3 (9.1)	1
Yes	55 (6.2)	828 (93.8)	2.69 (1.00-7.23)*	733 (83.0)	150 (17.0)	1.48 (0.50-4.28)	774 (87.7)	109 (12.3)	1.41 (0.42-4.69)
Methamphetamine									
No	50 (6.2)	762 (93.8)	1	681 (83.9)	131 (16.1)	1	724 (89.2)	88 (10.8)	1
Yes	10 (9.6)	94 (90.4)	0.62 (0.30-1.26)	81 (77.9)	23 (22.1)	1.48 (0.89-2.43)	80 (76.9)	24 (23.1)	2.47 (1.49-4.10)***
Other									
No	56 (6.3)	836 (93.7)	1	746 (83.6)	146 (16.4)	1	787 (88.2)	105 (11.8)	1
Yes	4 (16.7)	20 (83.3)	0.34 (0.11-1.01)*	16 (66.7)	8 (33.3)	2.56 (1.07-6.08)**	17 (70.8)	7 (29.2)	3.09 (1.25-7.62)**
Peer education in the past year									
No	45 (10.8)	373 (89.2)	1	325 (77.8)	93 (22.2)	1	363 (86.8)	55 (13.2)	1
Yes	15 (3.0)	483 (97.0)	3.89 (2.13-7.08)***	437 (87.8)	89 (12.2)	0.49 (0.34-0.69)***	441 (88.6)	57 (11.4)	0.85 (0.57-1.27)**
Unprotected commercial sexual in the past year									
No	52 (6.5)	752 (93.5)	1	680 (84.6)	124 (15.4)	1			
Yes	8 (7.1)	104 (92.9)	0.90 (0.42-1.95)	82 (73.2)	30 (26.8)	2.01 (1.27-3.18)**	—	—	—
Receptive sharing of syringe in the past year									
No	49 (6.4)	713 (93.6)	1						
Yes	11 (7.1)	143 (92.9)	0.89 (0.45-1.76)	—	—	—	—	—	—

*OR* Odds Ratio, *CI* Confidence Interval

*p < 0.10; **p < 0.05; ***p < 0.001 (p values for comparing the characteristics between IDUs are from Chi square test)

Older age, higher education, and peer education predicted higher level of HIV/AIDS knowledge awareness, while non-local residence, and ethnic minority predicted lower level of HIV/AIDS knowledge awareness. Non-local residence, ethnic minority and history of other drug use were risk factors for receptive sharing of syringes, while being recruited from community, female, higher education, and peer education predicted lower risk of receptive sharing of syringe. Sharing of syringes was also positively associated with inconsistent condom use. Non-local residence, history of methamphetamine use, and history of other drugs use were risk factors for inconsistent condom use, while, older age, being married or cohabiting, higher education, and peer education in the past year decreased the risk of inconsistent condom use.

### Multivariate Logistic Regression

Tables 3, 4, 5 shows the multivariate analysis of potential associated factors related to HIV/AIDS knowledge awareness, receptive sharing of syringes, and inconsistent condom use, respectively.

**Table 3:** Multivariate logistic analysis of the factors associated with HIV/AIDS-related knowledge awareness

	aOR (95 % CI)	*p* value
30-39 years age (vs.18-30 years age)	3.64 (1.69-7.83)	0.001
40-60 years age (vs.18-30 years age)	1.90 (0.90-4.01)	0.091
Non-local resident (vs. local resident)	0.25 (0.11-0.56)	0.001
Higher education level	2.01 (1.26-3.21)	0.003
Received peer education in the past year	2.87 (1.54-5.35)	0.001

*aOR* Adjusted Odds Ratio, *CI* Confidence Interval

**Table 4:** Multivariate logistic analysis of the risk factors associated with receptive sharing of syringe

	aOR (95 % CI)	*p* value
Han ethnic (vs. Minority)	0.29 (0.10-0.88)	0.029
Higher education level	0.64 (0.47-0.86)	0.004
Received peer education in the past year	0.54 (0.38-0.78)	0.001

*aOR* Adjusted Odds Ratio, *CI* Confidence Interval

**Table 5:** Multivariate logistic analysis of the risk factors associated with inconsistent condom use in commercial sex

	aOR (95 % CI)	*p* value
Married or cohabiting (vs. single)	0.20 (0.12-0.33)	<0.000
Divorced or widowed (vs. single)	1.33 (0.73-2.42)	0.350
30-39 years age (vs.18-30 years age)	0.69 (0.37-1.30)	0.254
40-60 years age (vs.18-30 years age)	0.37 (0.18-0.76)	0.006
Non-local resident (vs. local resident)	5.15 (2.29-11.56)	<0.000
Used methamphetamine in the past year	3.08 (1.64-5.81)	<0.000

*aOR* Adjusted Odds Ratio, *CI* Confidence Interval

Age 30–40 years (aOR = 3.64; 95 % CI: 1.69–7.83), non-local residents of Maanshan (aOR = 0.25; 95 % CI: 0.11–0.56), higher education (aOR = 2.01; 95 % CI: 1.26–3.21), and peer education (aOR = 2.87; 95 % CI: 1.54–5.35) were factors associated with HIV/AIDS knowledge awareness. Ethnic Han (aOR = 0.29; 95 % CI: 0.10–0.88), higher education (aOR = 0.64; 95 % CI: 0.47–0.86), and peer education in the past year (aOR = 0.54; 95 % CI: 0.38–0.78) were factors associated with receptive sharing of syringes. Married or cohabiting (aOR = 0.20; 95 % CI: 0.12–0.33), age 40-60 years (aOR = 0.37; 95 % CI: 0.18–0.76), non-local residents of Maanshan (aOR = 5.15; 95 % CI: 2.29–11.56), and history of methamphetamine use (aOR = 3.08; 95 % CI: 1.64–5.81) were factors associated with inconsistent condom use.

## Discussion

Our study found a relatively low prevalence of HIV infection among IDUs of whom were not previous HIV-positive diagnosis (0.33 %), which was similar to a previous study that investigated 385 community drug users (not previous HIV-positive diagnosis) in Maanshan city and found that the HIV infection rate was 0.78 % [10]. Maanshan is a small city located inland in the eastern Anhui province of China, and population mobility in Maanshan is very low. The local CDC had done extensive work to prevent HIV epidemic in recent years. Most IDUs in our study were male, which was similar to other cross-sectional studies among IDUs in Beijing [11] and South China [12]. We also found a higher level of HIV/AIDS knowledge awareness among participants in the current study. It was possible that IDUs were more aware of their susceptibility of contracting HIV or other sexually transmitted diseases (STDs), and hence they more actively participated in HIV prevention services. The other reason may be that IDUs were more likely to be the targeted for government-sponsored HIV education and intervention programs [13], through which they increased their HIV knowledge.

Our findings indicated that drug users in the 18-29 years age group reported lower level of HIV/AIDS knowledge awareness and higher level of inconsistent condom use in commercial sex as compared to older age groups. The possible reason is that as compared to older age groups, younger IDUs received less HIV/AIDS-related interventions and were more adventurous to engaged in high-risk behaviors such as injection drugs and unprotected commercial sex. Similarily, non-local residents reported lower level of HIV/AIDS knowledge awareness and relatively higher level of inconsistent condom use in commercial sex as compared to local residents. Non-local residents are much more mobile than local residents, which makes it difficult to administer harm-reduction programs to them. A previous study demonstrated that migrants lack access to public health services and are more likely to seek out commercial sex. [14] Our findings also revealed that individuals with higher education or those receiving peer education have higher odds of perceiving HIV/AIDS knowledge awareness, and lower odds of receptive sharing of syringe and inconsistent condom use. This may be because higher educated individuals have greater access to various HIV-related resources including the information, the access to condoms, and other harm-reduction materials and practices.

Our findings also indicated that ethnic minorities had higher odds of reporting receptive syringe sharing compared with ethnic Han. Illicit drug use, particularly heroin, has impacted ethnic minorities the hardest, with disproportionate numbers of HIV-infected drug users being of minority ethnicity [11]. Economic hardship, a history of tense relations with the Han-dominated government authorities, and poor access to healthcare services may be considered as drivers of drug use in ethnic minorities. And part of the general drug problem in China can be linked to its geographic proximity to the major opium producing regions in Southeast and Central Asia, where has led to availability of cheap drugs for ethnic minorities of whom living in the border region in southwest of China.

Our study revealed that married or cohabiting IDUs were more likely to use condoms in commercial sex during the past year which provided a low risk of HIV infection. Previous studies also found that the rate of condom use was lower among unmarried men than married men [15]. The higher prevalence of commercial sex behaviors and the lower frequency of condom use indicated a higher risk of disease from commercial sex among bachelors [16]. Therefore, it is reasonable to assume that a high prevalence of unprotected commercial sex among an increasing number of unmarried IDUs in China is likely to accelerate HIV/AIDS and STD transmission with a significant negative impact on public health. Unlike marital status, the use of methamphetamine prior to or during sex could increase the risk of unprotected sex practice among drug users [17, 18]. Several domestic studies indicated that drug users often have multiple sexual partners and are more likely to engage in unprotected sex [19], which leads to increased risk of transmission of HIV and other STDs in them. Therefore, public health professionals who develop syringe sharing reduction interventions among IDUs should also address risky sexual practices including inconsistent condom use [20].

The univariate analysis indicated that those IDUs who engaged in unsafe injecting practices also engaged in risky sexual practices, and this association may magnify the risk of HIV infection. Most interventions in China that aim to reduce HIV infection among IDUs have largely focused on transmission of infection through unsafe injecting drug use. However, there is also a need to understand and address sexual transmission of HIV infection among IDUs and their sexual partners. IDUs also have sexual intercourse with their wives without using condoms, and this might act as a bridge in transmitting the HIV from the pool of IDUs to wives of IDUs.

Several limitations of this study need to be considered when interpreting the findings. Firstly, our study population were recruited from MDC and community. Although the pooling of two samples whose characteristics are almost identical, there were still some potential bias which might influence our results. Secondly, we recruited drug users in the community through snowball sampling and our sample may not be representative of the general population of drug users in the Maanshan community. Thirdly, since drug use is illegal in China, IDUs may underreport their drug use activities. Information on personal and sensitive topics was collected through participants’ self-report, and our results may have been influenced by social desirability bias. Moreover, this study was conducted in Maanshan and our results may not reflect the risk behaviors of drug users in other Chinese cities. Systematic study of drug users in detoxification centers and the communities of other provinces is needed. Despite these limitations, the findings of the current study provide preliminary data for future research and interventions.

## Conclusions

There was a high level of HIV/AIDS knowledge awareness and relatively low level of unsafe injection practices and unprotected commercial sex behaviors among IDUs in the past year in Maanshan. Prevention programs targeting IDUs in Maanshan must also consider differences in demographic characteristics, and target ethnic minorities and less educated people to decrease unsafe injection practices such as sharing of syrings, and target non-local residents, younger and methamphetamine users to decrease unprotected commercial sex behaviors such as inconsistent condom use.
